# Optical Properties of *In Situ* Eye Lenses Measured with X-Ray Talbot Interferometry: A Novel Measure of Growth Processes

**DOI:** 10.1371/journal.pone.0025140

**Published:** 2011-09-20

**Authors:** Masato Hoshino, Kentaro Uesugi, Naoto Yagi, Satoshi Mohri, Justyn Regini, Barbara Pierscionek

**Affiliations:** 1 Japan Synchrotron Radiation Research Institute (SPring8), Sayo, Hyogo, Japan; 2 Kawasaki Medical School, Kurashiki, Okayama, Japan; 3 School of Optometry and Vision Sciences, Cardiff University, Cardiff, United Kingdom; 4 School of Biomedical Sciences, University of Ulster, Coleraine, United Kingdom; German Cancer Research Center, Germany

## Abstract

The lens, a major optical component of the eye, has a gradient refractive index, which is required to provide sufficient refractive power and image quality. The refractive index variations across the lens are dependent on the distributions and concentrations of the varying protein classes. In this study, we present the first measurements of the refractive index in the *in situ* eye lens from five species using a specially constructed X-ray Talbot grating interferometer. The measurements have been conducted in two planes: the one containing the optic axis (the sagittal plane) and the plane orthogonal to this (the equatorial plane). The results show previously undetected discontinuities and fluctuations in the refractive index profile that vary in different species. These may be linked to growth processes and may be the first optical evidence of discrete developmental stages.

## Introduction

Structure/function relationships are often described in terms of cells and their constituents. Shapes and physico-chemical characteristics of these small entities and sub-entities relate to their specific function and contribute to the function of the respective cell, tissue and organ. The links between structure and function can also be observed in nature on a larger organizational scale. This is particularly applicable to an organ such as the eye and its components. The function of the eye lens is optical. It needs to contribute sufficient refractive power to the eye so that light rays are directed to the photoreceptor cells in the retina. Fundamentally, it needs to be transparent. Both properties: refractive power and transparency depend on the material properties of the lens which are derived from the crystallin proteins and their proportional distributions across the tissue. The crystallins are broadly grouped into three classes in vertebrate lenses, designated α-, β- and γ-crystallin [1]. Other crystallin types have been identified in lenses of reptiles and amphibians [2]. However, regardless of crystallin types or proportions, lenses in all species investigated are transparent and have a refractive index gradient [3].

The reason for a gradient index lens in the eye is to provide a better optical performance than would be found with a homogenous index lens, as the former substantially reduces optical aberrations. The protein concentration and distribution patterns of each class are essential for creating the refractive index form and magnitude. The means by which this is maintained throughout life, especially given the continued growth of the lens, remains unknown. The exact form or shape of this gradient requires further investigation because it is linked closely with the lenticular mode of growth. The lens grows throughout life by accruing new cell layers over existing tissue, with no concomitant losses. Hence, every lens contains a chronological record of its development and growth processes and each layer of cells contributes to the shape of the refractive index profile. The index profile may therefore provide some insight into growth phases of the lens. This could eventually lead to a better understanding of the zones of discontinuity, lamellar-like features seen in the living human eye lens that may demarcate phases in lens development. To date there has been no clear explanation of the physical nature or purpose of these features. If these ‘zones’ are signs of protein density changes at certain life stages, they should appear as fluctuations or discontinuities in the refractive index profile. Studies on the refractive index of the lens, thus far, have been unable to detect such fine, localized fluctuations.

Previously, the profile shape of the refractive index distribution has, in a number of species, been approximated to a second order polynomial function. However, for human and some primate lenses the shape of the gradient is better fitted to a polynomial of higher order [3]. In addition to variations in the shape of the refractive index profile, differences exist in the magnitude of refractive index, across species. This depends on local protein concentration. It also depends on the differences in specific refractive increments of the various crystallins ie the contribution each class of proteins makes to the refractive index [4].

A number of techniques have been used to measure the index gradients of eye lenses. These range from methods that require tissue slicing [5], [6]; ray tracing [7]–[12], fibre optic sensing [13], [14] and magnetic resonance imaging [15], [16]. Ideally, the refractive index should be measured on whole lenses, in any given plane. Measurements should be made with a level of accuracy that can detect any fluctuations or irregularities in the profile that may be meaningful physiologically. X-ray microtomography allows for quantitative measurement of sample density but does not permit recognition of structures within the eyeball [17]. An X-ray Talbot grating interferometer, that combines phase contrast imaging and microtomography, has been developed by Momose [18], [19]. This interferometer makes use of the Moiré fringes generated by two gratings (an absorbance and phase grating). It also has the advantage, over interferometers such as the Bonse-Hart instrument, of being able to tolerate large density differences. This renders the technique applicable to the measurement of density changes within the eyeball. More specifically, it permits measurements of refractive index and protein density distributions across the eye lens as demonstrated using a murine lens [17]. This paper is the first study that has utilized the X-ray Talbot interferometer to measure protein concentrations and refractive index profiles in *in situ* lenses from five species. The aim of the study was to investigate whether this instrument could detect subtle changes in the refractive index gradient that could not be found with previously used techniques. Results indicate that subtle discontinuities or fluctuations in the index profile, which may have biological significance, can be detected using this method.

## Results

**Figure 1:**
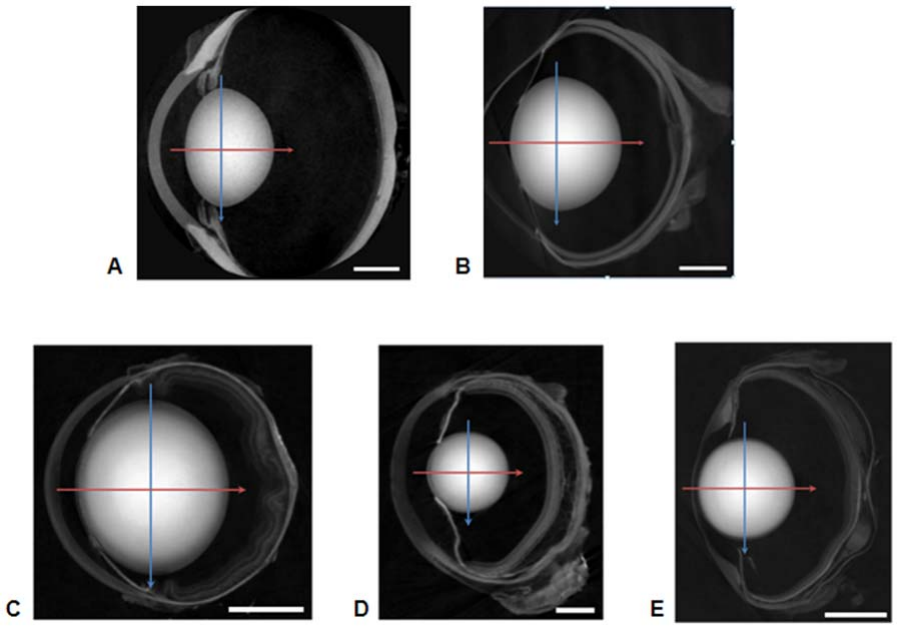
Images of a) porcine; b) ranine; c) murine; d) newt; e) piscine eyes in the sagittal plane. The position of the equatorial plane is marked with the blue arrow and the optic axis, along which the sagittal refractive index profiles were measured, is marked with a red arrow. The scale bars in the right hand lower corner are equal to a) 4 mm (porcine); b) 2 mm (ranine); c) 1 mm (murine); d) 0.5 mm (newt); e) 1 mm (piscine).

Images of the sagittal plane (the plane containing the optic axis) of the eyeballs from five species are shown in Figure 1. The lens is the prominent structural feature in each image. In the murine sample the ratio of lens/eyeball size is the largest (Figure 1c)) whereas in the porcine sample this ratio is the smallest (Figure 1a)). The newt and piscine lenses (Figures 1d) and e)) respectively are circular in the sagittal plane; the murine lens (Figure 1c)) is elliptical and the porcine (Figure 1a)) and ranine (Figure 1b)) lenses have asymmetric profiles with a greater curvature of the posterior than of the anterior surface. The image intensity varies across each lens and it is possible to discern regions of different density.

**Figure 2:**
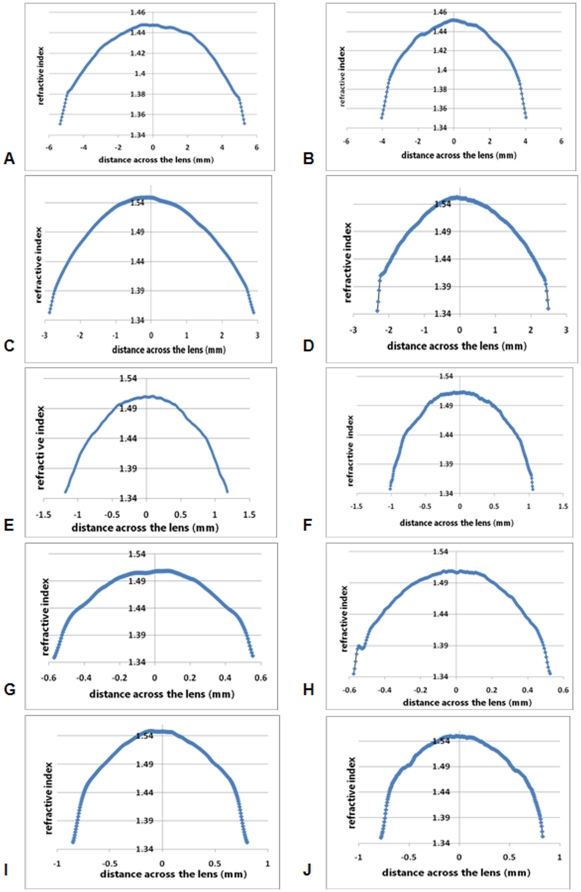
Refractive index profiles in two orthogonal sections of the equatorial plane of a) porcine; b) ranine; c) murine; d) newt; e) piscine lenses plotted against the distance across the lens in mm. Refractive index profiles along the optic axis (sagittal plane) of f) porcine; g) ranine; h) murine; i) newt; j) piscine lenses plotted against the distance across the lens in mm, from the anterior (-ve x-axis values) to the posterior (+ve x-axis values) poles.

Refractive index profiles measured in the equatorial plane of each lens are shown in Figure 2. All profiles have a minimal peripheral value of between 1.34 to 1.35 at the lens surfaces, rising to maxima of around 1.45 in the porcine lens (Figure 2a)) and just under 1.55 in the murine (Figure 2c)) and newt lenses (Figure 2d)). Whilst the profile shapes can be approximated to second order polynomials, there are ‘kinks’ or discontinuities in some of the functions where the curves deviate from second order polynomial fits. These kinks, seen in the peripheral sections of the profiles, are most prominent in the newt (Figure 2d)) and piscine lenses (Figure 2e)) and least obvious in the ranine lens (Figure 2b)). Table 1 shows the second order functions fitted to the refractive index profiles of lenses represented in Figure 2). These are shown in two orthogonal directions within the equatorial plane of each lens investigated.

**Table 1:** Second order polynomial functions fitted to refractive index profiles in the equatorial planes of lenses from five species.

Sample	Function fitted to equatorial profile (1)	Function fitted to equatorial profile (2)
Porcine	y = −0.003×^2^−0.0002×+1.4497 (R^2^ = 0.9883)	y = −0.0031×^2^−0.0001×+1.4518 (R^2^ = 0.9879)
Ranine	y = −0.0215×^2^−0.0019×+1.5496 (R^2^ = 0.9939)	y = −0.0211×^2^+0.0052×+1.5508 (R^2^ = 0.9951)
Murine	y = −0.1102×^2^+0.0017×+1.5128 (R^2^ = 0.9935)	y = −0.1112×^2^+0.0042×+1.5144 (R^2^ = 0.9936)
Newt	y = −0.4319×^2^+0.01×+1.5118 (R^2^ = 0.9821)	y = −0.4328×^2^−0.0037×+1.5123 (R^2^ = 0.9808)
Piscine	y = −0.2385×^2^−0.013×+1.552 (R^2^ = 0.9616)	y = −0.2393×^2^+0.01×+1.5529 (R^2^ = 0.9594)

Sagittal profiles from these lenses are shown in Figures 2f) to j). The porcine and ranine lenses have asymmetrical shapes in the sagittal plane. Whilst the sagittal refractive index profiles of the porcine lens reflect this asymmetry (Figure 2f)), it is far less evident in the ranine lens (Figure 2g)). The murine, newt and piscine lenses have approximately symmetrical refractive index profiles in the sagittal plane (Figures 2h), i) and j) respectively) although slight deviations from a smooth profile are seen. The murine, newt and piscine lenses are significantly smaller than the ranine and porcine lenses and, as all profiles are presented as comparably sized figures, deviations from a smooth profile will be more evident in smaller samples. It should be noted that the small peak in the anterior part of the newt lens profile arises because of a small opacity in that lens. The piscine lens profile (Figure 2j)) shows distinct discontinuities around 0.5 mm from the peak; a faint semblance of these can be seen in the equatorial profile (Figures 2e)).

**Figure 3:**
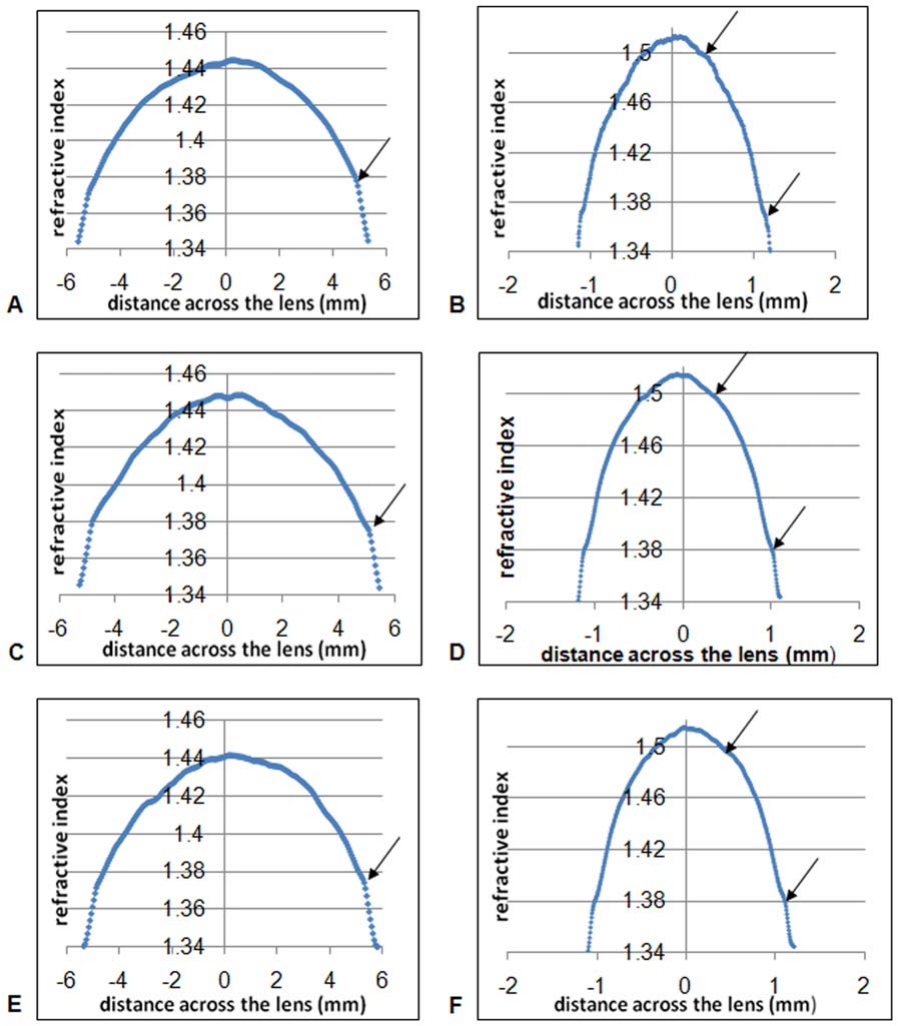
Refractive index profiles in the equatorial planes of three representative porcine (a),c) and e)) and three representative murine (b),d) and f)) lenses. Arrows point to discontinuities in the profiles.

In species for which more than one eyeball was measured, profiles were compared to see which types of deviations from a smooth profile appeared in different lenses from the same species. Figures 3 a), c) and e) show three representative porcine lens profiles. Arrows indicate the kink in the profile at around 0.5 mm from the outer edge of the lens that is a consistent feature in all of the porcine lenses examined. Murine lens profiles (Figures 3b), d) f)) show a definite discontinuity at around 0.1 mm from the edge of the lens and a slight indentation at around 0.3–0.4 mm from the lens centre.

**Figure 4:**
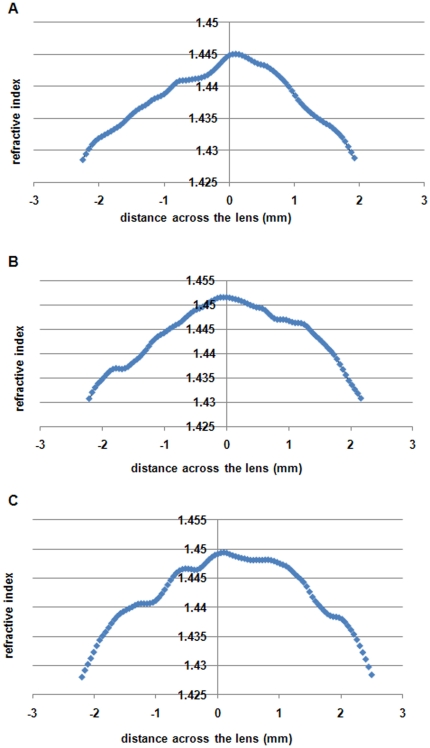
Central region of refractive index profiles along the optic axis (sagittal plane) in three porcine lenses plotted against the distance across the lens in mm, from the anterior (-ve x-axis values) to the posterior (+ve x-axis values) poles.

The most irregular sagittal profile is that of the porcine lens and this is particularly evident when the central most part of the porcine profile is highlighted. Figure 4 shows the region of the profile ±2 mm from the peak for three porcine lenses. All the figures show that from the central peak, which corresponds to the position of the equatorial plane, the refractive index slopes down less steeply on the anterior side compared to the posterior side of the lens. In addition, there are step-like fluctuations in the profiles.

**Figure 5:**
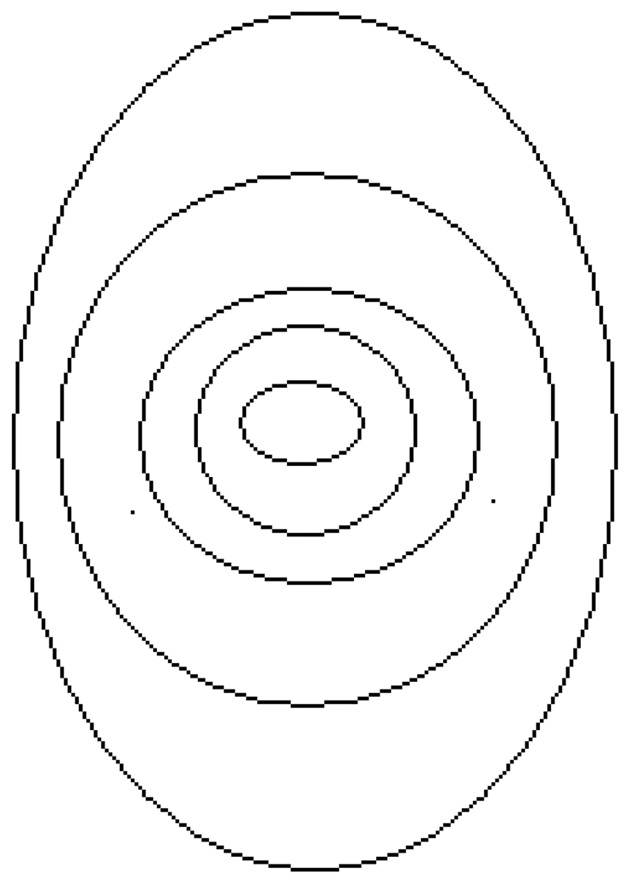
Pictorial representation of isoindicial contours of refractive index in a lens where the contours in the inner regions of the lens are wider in the sagittal than in the equatorial plane.

To determine whether the sagittal and equatorial profiles can be transposed directly onto one another, sections of the profiles from lenses shown in Figure 2, normalised to adjust for any differences in profile widths in the two planes, are compared in Table 2. The newt lens is the only one for which the proportions of the refractive index profile, above certain refractive index values, are the same for the equatorial as for the sagittal planes: eg the region with refractive index ≥1.50 is around 28% of the total profile in both planes. This suggests that in the newt lens, the refractive index is distributed along concentric, isoindicial contours that follow the surface shape. The piscine lens, the only other spherical lens examined, also has very similar proportions for given sections of the profiles in both planes (Table 2). There is greater variation in the non-spherical lenses, with the porcine lens showing the greatest differences in the proportional distribution of the refractive index values between the equatorial and the sagittal planes. The proportion of the profile with higher refractive index values is greater in the sagittal compared with the equatorial plane. In such a lens, the refractive index contours may be akin to those shown in Figure 5 (the figure is an approximation and does not show the asymmetry in surface shape).

**Table 2:** Widths of equatorial and sagittal refractive index profiles and the proportions of each profile with refractive index equal to and above a given value in lenses from five species.

Sample	Equatorial width (mm)	Sagittal width (mm)	Profile section	% of equatorial profile	% of sagittal profile
**Porcine**	11.04	8.06	≥1.44	32.17	37.50
			≥1.42	59.13	67.26
			≥1.40	73.91	83.93
**Ranine**	5.75	4.81	≥1.53	33.06	34.15
			≥1.51	46.12	48.29
			≥1.49	57.96	60.97
**Murine**	2.36	2.07	≥1.50	28.84	34.39
			≥1.48	46.28	53.70
			≥1.46	59.30	65.87
**Newt**	1.11	1.10	≥1.50	28.57	28.36
			≥1.48	48.77	48.26
			≥1.46	62.07	62.19
**Piscine**	1.65	1.60	≥1.53	33.89	37.20
			≥1.51	51.49	53.58
			≥1.49	63.79	65.19

Protein concentrations are linearly related to the refractive index by the Gladstone-Dale formula [20] and were calculated for 589 nm using a refractive increment of 0.18 ml/g. As the protein concentration can be calculated from the refractive index, the shapes of the protein concentration profiles are the same as those of the refractive index. The porcine lens has the lowest protein concentration: it reaches a maximum of around 0.75 g/ml in the central area of the lens. This compares to around 1.2 g/ml in the centres of ranine and piscine lenses and around 1 g/ml in the centres of the newt and murine lenses.

## Discussion

The measurement of refractive index in the eye lens has occupied scientists for centuries. In early attempts, the refractive indices of lens tissue samples were measured using Abbe refractometry [21], [22]. Subsequent measurements were made on slices or sections of tissue [5], [6]. These necessitated invasive procedures that would have altered tissue hydration and thereby reduced the accuracy of the results. Ray tracing methods offered the prospect of studying the refractive index variations in the intact eye lens. Following the seminal studies of Chu on optical fibre performs [23], these methods were applied to the rat lens [7], [8] and further tested on the lenses of various species [9]–[12]. Ray tracing alleviated the need to disturb tissue structure. However, the method required a mathematical treatise that assumed symmetry of shape and that isoindicial contours were concentric and followed the surface shape of the lens. For lenses with an asymmetric sagittal plane, ray tracing had to be conducted in the circularly symmetric equatorial plane, and the equatorial profile transcribed to the sagittal plane [24]. The other fundamental requirement for ray tracing analysis was that the lens surface index should be closely matched to the refractive index of the surrounding media. When matching with a physiologically suitable surround media was not possible, mathematical means were used to deal with discrepancies [25]. The aforementioned sources of inaccuracy notwithstanding, the general shapes of the index gradients and magnitudes over most of the profiles concurred with studies on water gradients [26] and protein concentration variations [27] across the lens.

A fibre optic reflectometer was constructed to enable localised refractive index measurements to be made on the lens surface and within any plane of the lens [13]. It offered the advantage of measuring refractive index directly at any point in the tissue. Although the method was invasive, the results were repeatable and were broadly comparable to the results of ray tracing [13], [14].

In more recent studies, magnetic resonance imaging was used to determine the refractive index in human and porcine lenses [15], [16]. However, this technique did not take into account changes in free and protein-bound water proportions that occur within the lens with age [28]. This led to conclusions of an age-related increase in overall water content in the lens. Such a process would require an imbibing of water by the lens or a loss of protein, neither of which could occur without disrupting the optical quality. Previous studies have found no change in the proportions of proteins and water in the human lens with age [29] beyond pre-natal and very early post-natal life [30].

Ideally the refractive index and the protein concentration of the lens should be measured on an intact sample maintained in a state that is as close as possible to its state in the eyeball. This avoids the sorts of changes that may lead to an alteration in hydration state and a consequent change in refractive index. Assumptions and interpretations applied should be consistent with what is known about the structure and physiology of the tissue. The X-ray Talbot grating interferometer used in this study offers, thus far, the most effective and accurate means of determining the refractive index in any plane or section of the lens without: removing it from the eyeball, necessitating assumptions about index contours or requiring a matching index surround.

The magnitudes of refractive index for the piscine and porcine profiles concur with earlier studies on similar species (*Carassius auratus auratus*) [9]; (*Sus domestica*) [31]. The murine (mouse) lens profiles show slightly lower protein concentration and index magnitudes than the earlier studies on the murine (rat) lens [7], [8]. It should be noted that previous studies used ray tracing methods with wavelengths around 633 nm. The refractive index profiles presented in this work were measured using X-ray energies between 15 and 35 keV (0.0827–0.0354 nm respectively). The comparable magnitudes suggest that there is little wavelength-related variation in the refractive index of the eye lens.

Lenses with steeper refractive index gradients and higher central index magnitudes have a greater proportion of the protein γ-crystallin [32]. This protein class has also been found to have the highest refractive increment [4] compared to the other crystallin proteins. The greatest refractive index maxima and the highest protein concentrations were found at the peaks of the ranine and piscine lenses. Accordingly, the centres of amphibian [32] and piscine lenses [33] have been found to contain only γ-crystallin.

Whilst the central (maximal) values of refractive index vary across species, there is less variation at the periphery. The refractive index magnitudes at the lens surface support previous work using both ray tracing and fibre optic sensing on human [24] and porcine lenses [31]. A surface index value that is not much higher than that of the aqueous allows for a greater proportion of the refraction to come from the gradient index within the lens rather than from the lens surface. As these samples have all been measured within the eyeball, there is no likelihood of any potential surface changes or dehydration that could result in an experimentally induced increase in the surface refractive index.

Only in spherical lenses (newt and piscine) did the refractive index profiles show similar distributions of refractive index in equatorial and sagittal planes (Table 2). This suggests that, within these lenses, the index contours may also be spherical. In the other lenses, the higher index regions were wider in the sagittal than in the equatorial plane even though the latter has the longer overall width. Although there is a general growth mode for the lens, there may be interspecies variations, particularly in early development, leading to differences in the shapes of the layers that contain the same protein concentrations (ie are isoindicial). Transposing refractive index measurements taken in one plane to another may therefore not be applicable in all lenses.

The refractive index profile for lenses of most species (that have thus far been studied) with the exception of human and higher order primates can be approximated to a second order polynomial [3]. However, Jagger [11] found, using eye models, that a second order polynomial gradient did not give the predicted image quality in a spherical fish lens and proposed a higher order polynomial fit of the form f(x) = a+bx^2^+cx^6^+dx^8^. Whilst second order polynomials may provide approximations to the refractive index profiles measured in this study, subtle deviations from a smooth function are evident.

The refractive index fluctuations found in this study are regular in some profiles and irregular in others. Clear discontinuities are seen in the sagittal plane of the piscine lens (Figure 2j)). These are approximately symmetrical, ie at the same distance from the equatorial plane and, suggest a change in the rate of protein synthesised at a certain stage of growth. Whilst these discontinuities were clearly visible in the piscine lens, a single lens is insufficient to conclude that these fluctuations are representative of that species and that they may be indicative of structural features. Certain consistencies were found, however, in the species for which more than one lens was available (porcine, murine). The murine and porcine lenses had distinct kinks in their profiles at around 0.1 mm and 0.5 mm from their respective lens edges (Figures 3). There was also a slight indentation within the murine lenses around 0.3–0.4 mm from the central section of the profile. These kinks and indentations suggest that the profiles are made up of sections which, from a structural perspective, may indicate natural discontinuities in growth, rather akin to the rings of a tree.

The porcine lens, which is the closest biochemically of all the species examined to the human lens, has the greatest irregularities in its profile around the peak region. Minor fluctuations in the three profiles, shown in Figure 4, vary but overall, there is a steeper gradient in the posterior compared to the anterior parts of both profiles. Whilst an asymmetry of lens shape is likely to lead to some asymmetries in the refractive index profile, it is not clear how this may be related to the irregularities seen in the peak profiles in Figure 4. The peak in each profile corresponds to the equatorial plane. This is the part of the lens where epithelial cells differentiate into the typical lens fibres that stretch from the equator to the anterior and posterior poles. The peaks of the profiles in Figure 4 also represent tissue that has been laid down in early gestation. The profile shapes suggests that the concentrations of proteins may not be evenly distributed along the fibre cells. It is not clear what optical advantage may be gained in the porcine eye by an asymmetry in the refractive index profile in the sagittal plane and particularly along the optic axis. These asymmetries are not seen in any of the other lens profiles. It should be noted that all the other lenses have higher refractive index magnitudes at the peak, signifying a greater protein concentration and tighter packing of cell constituents. Asymmetries may be less evident. Higher protein concentration also renders these lenses less compliant. Pliable lenses, like the human lens, alter their shape to adjust for variations in focussing distance whilst in animals such as mice, frogs or fish, such optical adjustment is not possible. Whilst is it not known whether the pig adjust its focussing by altering the shape of its lens, the porcine lens may be pliable enough to undergo some changes in shape, and hence internal tissue redistribution. This may account for some of the asymmetries in the profile.

The deviations from a smooth gradient that are seen in some of the profiles have not been reported in any previous studies. The mathematical analyses used in the ray tracing methods would have smoothed out such irregularities. Fibre optic sensing, which allows measurement of refractive index at specific points, may have omitted to find small localised fluctuations.

These features may suggest some changes in the growth mode of the lens, in its rate or in the complement of proteins laid down in the cells in the region of these irregularities. They may be manifestations of, what appear to be, layers of different protein density that have been labelled the zones of discontinuity [34]. Thus far these have only been observed in human lenses in the living eye. These zones of discontinuity do not affect refraction nor impair vision. They may, however, be indicative of important stages in the growth and development of the lens [34] and require further investigation. As each lens contains a chronological record of its growth, these processes can be studied in single lenses.

In conclusion, X-ray microtomography is able to detect subtle fluctuations in the index gradient that earlier methods have been unable to detect. This could provide very useful information about growth and development of the lens as well as insights into these processes for other organs. Such insights will, in turn, advance knowledge about biological function, life style requirements and optical performance of different species that could aid design of future optical systems.

## Methods

**Figure 6:**
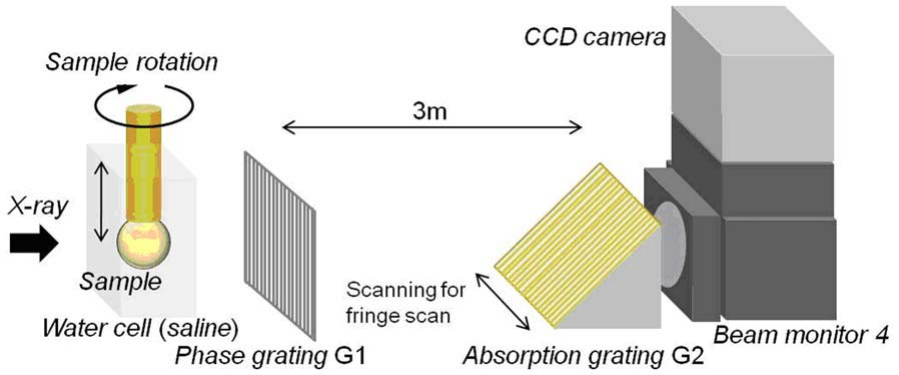
Diagrammatic representation of the X-ray Talbot interferometer showing the sample cell with sample suspended on a rotatable rod; the phase and absorption gratings (G1 and G2 respectively) and the beam monitor.

Experiments were conducted at the Japan Synchrotron Radiation Research Institute in the SPring-8 Synchrotron radiation facility at Hyogo, Japan. The X-ray grating interferometer, constructed at the bending magnet beamline BL20B2 in SPring-8, utilises a monochromatic X-ray beam that is passed through a Si(111) double crystal monochromator. The X-ray energy was tuned to 15 keV, 25 keV or 35 keV and the photon flux at an energy of 15 keV was 6.5×10^9^ (photons/sec/mm2@15 keV). The Talbot grating interferometer has two transmission gratings: a phase grating (G1) and an absorption grating (G2) (Figure 6). Grating parameters and materials were varied depending on the size of the lens samples. For large lenses grating G1 was made of tantalum and G2 was made of gold with pattern thicknesses 2.1 µm and 16.6 µm, respectively (Grating Set Type A in Table 3). The grating pitch of both gratings was 10 µm and the pattern size area was 25 mm (H)×25 mm (V). G2 was inclined by 45° so as to increase the effective X-ray absorption at the grating. For smaller samples, both gratings were made of tantalum and the pattern thicknesses of G1 and G2 were equal to 0.96 µm and 4.75 µm, respectively (Grating Set Type B in Table 3). The pitch of both gratings was 5 µm and the pattern size area was 5 mm (H)×10 mm (V). The inclination angle of G2 was 60°. An appropriate X-ray imaging detector (consisting of a beam-monitor and a charge coupled device (CCD) camera) was selected to acquire an image of the whole eyeball with an adequate field of view (Table 3). For large lenses (Grating Set Type A), the final field of view was determined by the detector; for smaller lenses (Grating Set Type B), the effective field of view was limited by the horizontal size of the gratings.

**Table 3:** Parameters used in the measurement of refractive index.

Sample	X-ray energy (keV)	GratingSet	Effective pixel size(µm)	FOV(H×V)(mm×mm)	Steps	Projections	Measurement time (min)
Porcine	35	Type A	48	24×24	5	600	35
Piscine	15	Type B	5.5	5.5×3.6	5	600	75
Murine	15	Type B	5.5	5.5×3.6	5	600	75
Ranine	25	Type A	23.4	23.4×15.4	3	900	25
Newt	15	Type B	5.5	5.5×3.6	5	600	75

FOV: Effective field of view of detector; Steps: Number of scanning steps of G2 in a period of the visibility curve to retrieve the phase shift; Projections: Number of projections acquired in 180 degrees rotation of a sample for X-ray phase contrast tomography.

Phase retrieval was achieved using a fringe-scan method [18], [19]. G2 was shifted with a Piezo stage (for Grating Set B) or a motorized stage (for Grating Set A) and either 5-step or 3-step fringe-scans were used (Table 3). Differential phase shift images were obtained and integrated to provide the phase shift image. The 3-step fringe scan (ie 3 images required for phase retrieval) was used on one of the samples (*Rana catesbeiana*) (Table 3) to ascertain whether the comparatively decreased measurement time of the 3-step fringe scan would result in any notable reduction in the phase contrast image quality.

Lenses from five species: pig (*Sus domestica*) (5), fish (*Carassius auratus auratus*) (1) mouse (*C57BL/6*) (4), frog (*Rana catesbeiana*) (1) and newt (*Cynops pyrrhogaster*) (1) were examined within intact fresh eyeballs using the X-ray grating interferometer. All eyeballs were obtained in Japan: the porcine samples from the local abattoir, piscine and newt samples from local pet shops, ranine samples from the Ouchi Frog Farm in Saitama and murine samples from Japan SLC Inc. Ethical approval for use of these samples was granted by the Animal Ethics Committee of SPring-8. Eyeballs were attached to a perspex rod that was suspended in saline (1.006 g/cm^3^) within a specially constructed cell (Figure 6). For large lenses, the X-ray energy was increased to obtain adequate X-ray transmission. Measurement conditions for each lens are shown in Table 3. The phase shift was calibrated against five solutions of known density: water, normal saline of 1.006 g/cm^3^ and salt solutions of 1.051 g/cm^3^, 1.110 g/cm^3^ and 1.143 g/cm^3^ and the theoretically obtained values were compared to the experimentally derived phase shift values per pixel. The relationship was found to be linear over the range of concentrations tested.

The following equation was used to convert the phase shift value/pixel (ΔΦ) to the X-ray refractive index difference from saline in the cell (Δδ):
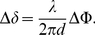
(1)where

λ is the X-ray wavelength

d is the pixel size.

The X-ray refractive index difference, Δδ, was used to estimate the crystallin concentration in the lens assuming that the lens is composed of crystallin proteins and water.

The number of electrons included in unit volume N (e^−^/ml) is:
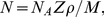
(2)where

N_A_ is Avogadro's constant,

Z is the number of electrons or atomic number,

ρ is the density in g/ml and

M is the molecular weight.

Taking the volume fractions of water F_w_ and crystallin F_cry_:
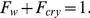
(3)If the number of molecules included in unit volume of water and crystallin is defined as:
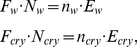
(4)where

n_w_ is the number of molecules in a unit volume of water and,

n_cry_, is the number of molecules in a unit volume of crystallin protein,

E_w_ (e^−^/mol) is the number of electrons in a single molecule of water and

E_cry_ (e^−^/mol) number of electrons in a single molecule of crystallin protein.

The number of electrons, N_exp_, in a protein solution of 1 ml is estimated from the phase contrast CT image. The difference in the number of electrons per unit volume between the protein and the saline solutions (ΔN) equals
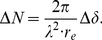
(5)where

r_e_ is the classical electron radius.

The total number of electrons N_exp_ equals:
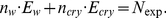
(6)and
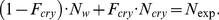
(7)The volume fraction of crystallin can be calculated from equation 7) and used to obtain the density of the crystallin solution ρ_sol_:
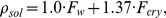
(8)where the protein density equal to 1.37(g/ml) is estimated using a partial specific volume of protein = 0.73. The crystallin concentration ρ_cry_ (g/ml) equals:
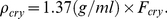
(9)

